# A Manual of Medicine

**Published:** 1902-12

**Authors:** 


**A Manual of Medicine.** Edited by W. H. Allchin, M.D., F.R.C.P., F.R.S. Vol. III. "Diseases of the Nervous System." London: Macmillan and Co. 1901.

The review of this excellent volume is long overdue, but a brief reference to its good qualities cannot be omitted. It is well up to the standard set in the two earlier volumes. There is perhaps more excuse here for the large proportion of the Editor's limited space, which is taken up by chapters on Anatomy and Physiology. Indeed, Sherrington's introduction to the Physiology of the Nervous System is so condensed that, able as it is, the meaning and importance of the statements are not always apparent to the average reader.

Dr. William Aldren Turner occupies about a quarter of the book with articles on the Neurone in relation to Disease of the Nervous System and Organic Diseases of the Brain and Membranes. These subjects are beautifully arranged, and tabulated in a helpful manner. Indeed, one of the chief features of the volume is the excellent way in which recent pathology is employed to explain the relations of the various affections of the nervous system to each other, and the care with which the differential diagnosis is worked out. The classification of acute meningitis follows that of Osler; but the writer confesses the difficulty of choosing a satisfactory arrangement, and has to go over some subjects which have been already treated in an earlier volume. Some readers may perhaps object to the statement that the most common cause of embolism is pyœmia with ulcerative endocarditis, though it is certainly an important one. An interesting article of some length is that by Collier on Defects of Speech, which are carefully classified, and their causation is well worked out. After two more chapters on the Morbid Anatomy of the Cord and the general symptomatology, we get Dr. Ormerod's discussion on Diseases of the Substance of the Cord, divided into system and non-system ones. The motor paralyses are grouped as spastic with degeneration of the upper neurons, and placcid from affection of the lower one.

Epilepsy, hysteria, and chorea, will be found some of the most interesting articles in the section on Functional Diseases. We notice that Poynton and Payne's recent explanation of rheumatic chorea is mentioned as an important advance in a difficult subject.

It seems a pity that two articles on Medical Ophthalmology and Electricity should take up space which would have been well devoted to more detailed accounts of various diseases; but it must be confessed that wonderful skill has been exercised in framing such clear and instructive descriptions of each disease. The fact that certain affections generally grouped together are broken up into separate headings makes the descriptions seem briefer than they really are.

